# The Possible Mediatory Role of Inflammatory Markers on the Association of Dietary Insulin Index and Insulin Load with Metabolic Syndrome in Women with Overweight and Obesity: A Cross-Sectional Study

**DOI:** 10.1155/2023/1979124

**Published:** 2023-08-21

**Authors:** Melika Darzi, Farideh Shiraseb, Alessandra da Silva, Josefina Bressan, Cain C. T. Clark, Sara Mahmoodi, Khadijeh Mirzaei

**Affiliations:** ^1^Department of Nutrition, Science and Research Branch, Islamic Azad University, Tehran, Iran; ^2^Department of Community Nutrition, School of Nutritional Sciences and Dietetics, Tehran University of Medical Sciences (TUMS), Tehran, Iran; ^3^Department of Nutrition and Health, Universidade Federal de Vicosa, Vicosa, Minas Gerais, Brazil; ^4^Centre for Intelligent Healthcare, Coventry University, Coventry, CV1 5FB, UK; ^5^Department of Clinical Nutrition, School of Nutritional Sciences and Dietetics, Tehran University of Medical Sciences (TUMS), Tehran, Iran; ^6^Food Microbiology Research Center, Tehran University of Medical Sciences, Tehran, Iran

## Abstract

**Background:**

Metabolic syndrome (MetS) is associated with increased inflammation. Diet plays an important role in the prevention and management of MetS, while some dietary factors can also increase or decrease markers of systemic inflammation. In this study, we aimed to determine the mediated association of inflammatory markers induced by dietary insulin index (DII) and dietary insulin load (DIL) with MetS and its components.

**Methods:**

This cross-sectional study was conducted with 219 women aged 18–28 years. Dietary intake was assessed by a 147-item food frequency questionnaire (FFQ). DII and DIL were calculated using the standard formula. The guidelines of the National Cholesterol Education Program's Adult Treatment Panel III were used to define MetS. Biochemical parameters and anthropometric and blood pressure measures were evaluated by standard protocols.

**Results:**

After the adjustment for potential confounders, a marginally significant association was found between DII and MetS (OR = 2.11; 95% CI = 0.93–4.82; *P* = 0.06). However, we did not find a significant association between DIL and MetS. Furthermore, DII was significantly associated with waist circumference (WC) (OR = 1.67; 95% CI = 1.09– 4.03; *P* = 0.03) and marginally associated with triglyceride (TG) (OR = 1.10; 95% CI = 0.92–2.33; *P* = 0.07) and systolic blood pressure/diastolic blood pressure (SBP/DBP) (OR = 1.84; 95% CI = 0.85–3.99; *P* = 0.07). Moreover, there was a significant association between DIL and SBP/DBP (OR = 1.74; 95% CI = 1.54–5.61; *P* = 0.04). Also, we found that MCP-1 may have a mediatory role in the association between DII and DIL with MetS and several components of MetS. Hs-CRP did not have mediatory role in the association between DII and MetS. However, hs-CRP had a mediatory role in several MetS components. Furthermore, hs-CRP may have a mediatory role in the association of DIL with MetS and with some of its components.

**Conclusions:**

A higher DII score may increase the odds of MetS and its components. DIL was not significantly associated with the odds of MetS, but the association of DIL and SBP/DBP was significant. MCP-1 may have a mediatory role in associations between DII and DIL with MetS. In addition, hs-CRP may have a mediatory role in the association between DIL and MetS.

## 1. Introduction

Metabolic syndrome (MetS) is defined by the presence of at least three metabolic risk factors at the same time, including impaired glucose metabolism, abdominal obesity, hypertension, high triglyceride levels, and low high-density lipoprotein (HDL) levels [1]. The global prevalence of MetS in the adult population has been reported to be between 20% and 25% [2]. Moreover, 34.8% of women and 25.7% of men in Iran are estimated to suffer from metabolic syndrome [3]. MetS can potentially increase the risk of type 2 diabetes mellitus (T2DM), cardiovascular disease (CVD), and related mortality [4] and, therefore, is considered to be a global public health concern [5]. The precise underlying cause of MetS is still unclear. However, many factors and mechanisms such as hyperinsulinemia, obesity, adipose tissue disorders, inflammation, and oxidative stress have been implicated in the development of the MetS [6]. Lifestyle modification, including dietary changes and increasing physical activity, is the first-line therapy for MetS [7].

Several dietary factors have been associated with a higher risk of MetS [8–10]. Indeed, recent studies have shown that diets that induce higher postprandial insulin secretion may gradually decrease the function of pancreatic *β* cells, which may result in glucose intolerance and increase the risk of chronic diseases [11]. The dietary insulin index (DII) represents the postprandial insulin response in comparison with isoenergetic reference food (glucose or white bread) [12]. This index is more accurate than the glycemic index because, in addition to carbohydrates, it also considers other dietary factors, such as proteins or fats that can stimulate insulin secretion [13]. Dietary insulin load (DIL), which is based on DII, is calculated by multiplying the DII of each food by its energy content and frequency of consumption [14].

Diets with a higher insulin index may be associated with inflammation and inflammatory biomarkers [15, 16]. For instance, elevated high-sensitive C-reactive protein (hs-CRP) and tumor necrosis factor receptor 2 (TNFa-R2) levels were seen in participants with higher insulinemic diets [17]. However, in another study, there were no significant associations between the DII and DIL with inflammatory biomarkers, including IL-6 and CRP [18]. Inflammation might have a triggering factor for the development of the metabolic syndrome and its components [19]. Studies have shown an association between levels of inflammatory biomarkers and metabolic syndrome [20–22]. A cross-sectional study reported that the concentrations of hs-CRP, IL-6, and MCP-1 were significantly higher in patients with MetS [21].

Studies that have evaluated the association among DII, DIL, and MetS are inconsistent. Sadeghi et al. observed a positive association between higher DII and DIL with MetS [23], whereas another cross-sectional study did not find any significant association [24]. Moreover, to our knowledge, no study has examined the mediating relationship between inflammatory biomarkers and DII and DIL with MetS. Therefore, this study sought to investigate the mediatory role of inflammatory markers (hs-CRP and MCP-1) on the association between DII and DIL with MetS in women with overweight and obesity.

## 2. Methods and Materials

### 2.1. Study Population

Two hundred nineteen healthy women with overweight and obesity of reproductive age (18–48 years) were recruited in the present cross-sectional study in 2018. The qualified participants were enrolled using multistage cluster random sampling in healthcare centers in Tehran, Iran. Healthy women were enrolled in the study with no specific diets and a body mass index (BMI) of 25–40 kg/m^2^. All forms of chronic disease, consumption of alcohol or any medication (including oral contraceptives), noticeable weight changes (more than 10%) in the last 6 months, pregnancy, lactation, menopause, smoking, and underreported (>800 kcal/d) or overreported (>4200 kcal/d) total calorie intake were considered as noninclusion criteria in our study [25]. Before being recruited for the study, each person provided the written informed consent. The protocol of this study was accepted by the ethical committee of the Tehran University of Medical Sciences (TUMS).

### 2.2. Anthropometric and Body Composition Assessments

With a nonstretchable tape, the subjects' height was measured to the nearest 0.1 cm. The waist circumference (WC) was also measured at the midpoint between the last rib and the iliac crest. Body weight (kg), BMI, and body composition, including fat-free mass (FFM) and body fat mass (BFM), were assessed by a bioelectrical impedance analyzer (In Body 770 scanner, Seoul, Korea). According to manufacturer guidelines, participants had light clothes and took off their shoes, socks, and any metal objects.

### 2.3. Dietary Assessment and Calculation of DII and DIL

An annual semiquantitative food frequency questionnaire (FFQ) was used to assess participants' regular dietary intake [26]. The FFQ contained 147 items with standard serving sizes usually consumed by the Iranian population. The validity and reliability of FFQ have already been verified [27]. Each food item's frequency of intake was divided into daily, weekly, monthly, and yearly categories. Finally, the reported frequencies for each item were converted to a daily intake. Each food serving size was converted from household measurements to grams [25]. Face-to-face interviews with a trained nutritionist were used to administer the FFQ. Nutritionist IV software was used to calculate total energy, macro, and micronutrients.

The insulin index of food is the difference between the area under the insulin curve after consuming a 1,000 kJ (239 kcal) portion of the test food divided by the area under the curve after consuming an isoenergetic portion of the reference food over two hours. The insulin index was gathered from earlier research by Brand-Miller [12]. For food items in which insulin indexes were not found in the reference food lists, insulin indexes from similar food items were used. The insulin load of each food item was computed by the following formula:

(1) $$\begin{array}{c} insulin\;load\;of\;food=insulin\;index\;of\;food\times energy\;content\;per\;1\;gram\;of\;food\;\left(kcal\right)\times amount\;of\;food\;consumed\left(\frac{g}{d}\right)\text{.} \end{array}$$

DIL for each subject was calculated by the sum of the insulin loads of all food sources in FFQ. After that, DII was determined for each individual by dividing DIL by total caloric intake [28].

### 2.4. Biochemical Assessment

After overnight fasting, blood samples were taken to the nutrition and biochemistry laboratory of the Tehran University of Medical Sciences. The samples were stored at –80 Celsius. Serum concentrations of insulin were analyzed by the enzyme-linked immunosorbent assay (ELISA) method (Human insulin ELISA kit, DRG Pharmaceuticals, GmbH, Germany), and fasting blood glucose (FBG) was measured through the glucose oxidase method. Insulin resistance was assessed by the homeostasis model assessment (HOMA): insulin (µU/mL) × fasting glucose (mmol/L)/22.5. Triglyceride (TG) was evaluated with triacylglycerol kits (Pars Azmoon Inc., Tehran, Iran) by using the glycerol-3-phosphate oxidase phenol 4-aminoantipyrine peroxidase (GPOPAP) method. Total cholesterol (TC) levels were assessed with the Enzymatic Endpoint method. HDL and low-density lipoprotein (LDL) were measured by an enzymatic clearance assay. Hs-CRP was measured by an immunoturbidimetric test with the Pars Azmoon kit. The ELISA method was also used for measuring MCP-1 levels.

### 2.5. Assessment of Metabolic Status

MetS was defined according to the National Cholesterol Education Program's Adult Treatment Panel III (ATP-III), which included the presence of at least 3 of the following criteria: abdominal obesity (WC ≥ 88 cm in women), altered fasting blood glucose (FBG level ≥100 mg/dL), hypertension (blood pressure >130/85 mmHg), and hyperlipidemia (HDL <50 mg/dl and triglycerides >150 mg/dl) [29]. BMI was determined according to the World Health Organization (WHO): overweight ≥25 kg/m^2^ and obesity ≥30 kg/m^2^ [30].

### 2.6. Assessment of Blood Pressure

Blood pressure was measured by a trained physician after at least 10 minutes of rest in a sitting position using a standard sphygmomanometer (Omron, Germany, and European). Three measurements at five-minute intervals were taken.

### 2.7. Assessment of Other Variables

Data on physical activity were estimated using the International Physical Activity Questionnaire (IPAQ), and the metabolic equivalent (MET-minutes/week) was then calculated for each subject [31]. Age, marital status (single, married), economic (low, moderate, and high), and educational levels (illiterate, under diploma, diploma, bachelor, and higher) were collected by a demographic questionnaire.

### 2.8. Statistical Analysis

The Kolmogorov–Smirnov test was used to evaluate the normality of dependent quantitative variables. Quantitative and categorical variables were presented as the mean (standard deviation (SD)) and absolute frequency (percentage), respectively. Subject characteristics, anthropometric variables, biochemical markers, body composition, and dietary intake were categorized based on the median DIL (97155.69) and DII (38.82) scores. Quantitative variables according to categories of DIL and DII were evaluated through one-way analysis of variance (ANOVA), whilst for categorical variables, chi-square (*χ*^2^) was applied. Moreover, analysis of covariance (ANCOVA) was used to adjust potential confounders. Binary logistic regression was used to estimate the association between DIL and DII with MetS and its components. Odds ratios (OR) and 95% CI were reported accordingly. The lower median of DIL (<97155.69) and DII (**<**38.82) were considered as reference groups. Model 1 was adjusted for BMI, age, energy intake, and physical activity, and model 2 was adjusted for model 1 further with education, marital status, and economic status. Hs-CRP and MCP-1 were entered separately as confounding variables in the final model for investigating the mediatory role of inflammatory biomarkers. SPSS software (version 26.0; SPSS Inc., Chicago, IL, USA) was used for statistical analyses, where *P* < 0.05 was considered significant and *P* = 0.05, 0.06, and 0.07 were reported as marginally significant.

## 3. Results

### 3.1. Study Population Characteristics

The mean age of 219 women who participated in this study was 36.67 (9.10) years, and the means of anthropometric characteristics such as height, BMI, weight, and WC were 161.22 (5.87) cm, 31.26 (4.29) kg/m^2^, 81.29 (12.43) kg, and 99.61 (10.07) cm, respectively. The means of DII and DIL were 39.78 and 105070.52. Moreover, the means DBP, SBP, TG, FBS, and HDL were 77.60 (10.40) mmHg, 111.38 (14.80) mmHg, 118.10 (24.12) mg/dL, 87.42 (9.64) mg/dL, and 46.58 (10.86) mg/dL, respectively. The means of two inflammatory biomarkers hs-CRP and MCP-1 were 4.34 (4.62) mg/L and 49.29 (15.40) mg/dl. Most participants were married (72.4%) and had an academic education (47.8%).

### 3.2. Description of General Participant Characteristics among Median DIL and DII

Women were categorized among the median DII and DIL. General characteristics of subjects such as anthropometric, body composition, biochemical, and other variables among lower vs. higher than the median of DII and DIL are shown in Table 1. In the crude model, participants in the lower median group of DII had significantly higher economic status (*P* = 0.04) compared to the higher median group of DII. In addition, participants with the higher median of DIL had significantly higher fat-free mass (*P* = 0.03). After adjusting for age, BMI, energy intake, and physical activity, the results showed that participants with the lower median of DIL had significantly higher education level than participants with the higher median of DIL (*P* = 0.02).

### 3.3. Description of Dietary Intakes of the Study Population between the Median of DII and DIL

The subjects' dietary intake among the medians of DII and DIL is shown in Table 2. After adjustment for energy intake, the results showed that participants with the higher median of DII significantly had higher intake of fruits (*P* = 0.01), carbohydrate (*P* ≤ 0.001), iron (*P* = 0.009), magnesium (*P* = 0.004), phosphorus (*P* = 0.01), and thiamin (*P* ≤ 0.001). In contrast, they significantly had lower intake of legumes (<0.001), meat (<0.001), fat (<0.001), polyunsaturated fatty acid (PUFA) (<0.001), and vitamin E (<0.001). Also, participants with the higher median of DIL significantly had higher intake of vegetables (*P* = 0.04), nuts (*P* = 0.02), carbohydrate (*P* = 0.01), fat (*P* = 0.005), protein (*P* = 0.001), PUFA (*P* = 0.004), vitamin E (*P* = 0.03), and thiamin (*P* = 0.001) compared with those with the lower median of DIL.

### 3.4. Association of DIL and DII with MetS and Its Components

The association between MetS and its components with DIL and DII is presented in the crude and two adjusted models by binary regression in Table 3. In the crude model, we did not observe any significant association between DII and DIL with MetS. However, women with a higher median of DII tended to have 2.12-fold higher odds of high SBP/DBP compared with women in the lower DII (OR = 2.12; 95% CI = 1.10; 4.11; *P* = 0.02). Moreover, women in the higher median of DII had 88% greater odds for high DBP (OR = 1.88, 95% CI = 0.95; 3.71, *P* = 0.06). In model 1 after controlling for age, BMI, energy intake, and physical activity, we did not observe any significant association between DII and DIL with MetS and its components. In model 2, after adjusting for model 1 confounding variables in addition to education, marital, and economic status, a marginally significant association was seen between DII and MetS, such that women in the higher median of DII tended to have 2.11-fold higher odds for MetS compared with those in the lower median of DII (OR = 2.11, 95% CI = 0.93; 4.82, *P* = 0.06). Moreover, we found that women in the higher median of DII had 67% increased odds of high WC compared to the lower median of DII (OR = 1.67; 95% CI = 1.09–4.03; *P* = 0.03). Furthermore, marginally significant associations were seen between DII with TG and SBP/DBP, such that women with the higher median of DII had greater odds for high TG (OR = 1.10; 95% CI = 0.92–2.33; *P* = 0.07) and high SBP/DBP (OR = 1.84; 95% CI = 0.85–3.99; *P* = 0.07). No significant association was seen between DIL and the risk of MetS. Nevertheless, in model 2, women with the higher median of DIL had 74% greater odds for high SBP/DBP (OR = 1.74; 95% CI = 1.54–5.61; *P* = 0.04) compared with those with the lower median of DIL. However, no significant association was found between other components of MetS and DIL.

### 3.5. Mediatory Role of Inflammatory Biomarkers on Association between DIL and DII with MetS

We evaluated the role of inflammatory biomarkers, including hs-CRP and MCP-1, as mediatory markers for the association between DII and DIL with MetS and its components (Table 4). When MCP-1 was entered into the final model adjustment in DII groups, the significance in WC, TG, HDL, FBG, and MetS decreased. Therefore, the results suggest that MCP-1 might be considered a mediatory marker in WC (*P* = 0.43), TG (*P* = 0.49), HDL (*P* = 0.98), FBG (*P* = 0.66), and MetS (*P* = 0.11). The significance was decreased in WC (*P* = 0.28), TG (*P* = 0.60), SBP (*P* = 0.76), DPB (*P* = 0.31), and SBP/DBP (*P* = 0.13) when hs-CRP was included as confounding variable and was indicative of being a mediatory marker in these variables. However, the results also showed that hs-CRP might not be considered a mediatory marker in MetS. In DIL, results suggested that both inflammatory markers MCP-1 (*P* = 0.53) and hs-CRP (*P* = 0.50) might have mediatory roles in MetS. MCP-1 also had mediatory roles in HDL (*P* = 0.90), SBP (*P* = 0.51), and SBP/DBP (*P* = 0.20), and hs-CRP had mediatory roles in WC (*P* = 0.20), DBP (*P* = 0.36), and SBP/DBP (*P* = 0.53).

## 4. Discussion

In the current cross-sectional study, we evaluated the possible relationship between DII and DIL with MetS, mediated by inflammatory markers, including hs-CRP and MCP-1. To our knowledge, this is the first study to evaluate the mediatory role of inflammatory markers regarding this association. Few studies have assessed the association between DII and DIL with MetS, and the results have been controversial, whilst the mediatory role of inflammatory markers has not been considered [23, 24]. We found that hs-CRP had a mediatory role in the associations between DII and DIL with several MetS components, such as WC, TG, SBP, DBP, and SBP/DBP. Also, the results suggested that MCP-1 likely has a mediatory role in MetS and several other components, including WC, TG, HDL, FBG, and SBP/DBP.

Dietary habits that elicit an increased insulin response may contribute to the development of obesity and fat storage [32]. This can increase various cytokines and inflammatory biomarkers and promotes systemic inflammation [33]. For instance, studies have found that MCP-1 protein expression was higher in adipose tissues of patients with obesity, and the circulating MCP-1 becomes increased by high-glycemic index diets [34, 35]. Furthermore, studies have investigated the association between some inflammatory markers and MetS and its components. It has been suggested that high CRP levels may be strongly associated with central adiposity, insulin resistance, blood pressure, high TG, and low HDL [36–38]. Further, several studies have reported that MCP-1 was higher in individuals with MetS [39].

In addition, hyperinsulinemia, which is positively associated with diets with high insulinemic foods, can influence the function of pancreatic *β* cells and gradually increase *β* cell death. As a result, macrophages infiltrate into pancreatic islets and produce proinflammatory cytokines, and this can be the onset of glucose intolerance and type 2 diabetes [40]. Indeed, empirical investigations support the hypothesis that inflammatory markers may have a mediatory role in the initiation and progression of MetS.

Our findings showed a significant association between DII and WC, and marginally significant associations were seen in TG, SBP/DBP, and MetS. Regarding DIL, there was no significant association between MetS and DIL, but a significant association was seen in SBP/DBP after adjusting for potential confounders.

Recent studies have assessed the association among DII, DIL, and metabolic risk factors [14, 41–43]. A cross-sectional study demonstrated that women who followed a diet with a high DII had a higher risk of developing general obesity [32]. Moreover, in accordance with our study, positive associations were found among DII, WC, and MetS [41]. The possible mechanisms for the association between DII and general or abdominal obesity might be related to the effects of insulinogenic foods that cause reduced insulin sensitivity and higher insulin secretion, which can increase glucose uptake and lipogenesis. This would conceivably elevate body fat, particularly in the abdominal area. Furthermore, high insulinemic foods are digested and absorbed immediately, and a quick drop in blood glucose induces the feeling of hunger and causes increased calorie intake and obesity [44, 45]. Also, some studies assessed the relationship between DII/DIL and glycemic status, and, in contrast with our study, Mozaffari et al. found a positive association between DIL and FBG [15]. According to another study, higher DII and DIL are associated with the greater development of insulin resistance [14]. A high intake of insulinemic foods may progressively reduce the function of pancreatic *β* cells, which causes insulin resistance [46]. However, in another study, DII and DIL were not associated with markers of glycemic control [18]. In the present study, we noted a marginally positive significant association between DII and TG. In agreement with our study, Nimptsch et al. reported that higher DII was associated with higher TG concentrations and lower HDL [18]. However, another study reported no relationship between dietary insulin indices and lipid profiles [15]. As previously mentioned, dietary insulin index and load are related to insulin resistance and can result in lower HDL and reduced function of lipoprotein lipase. This increases the synthesis of free fatty acids from adipocytes and causes the production of TG [47]. Moreover, we found a significant positive association between DII and DIL and SBP/DBP. Previous studies, to our knowledge, had not investigated this association. One of the possible explanatory mechanisms is related to the effect of DII and DIL on insulin resistance. Indeed, vasodilator nitric oxide (NO) is stimulated by insulin, and in a healthy state, the release of insulin after eating causes the skeletal-muscle vasculature to dilate, but in conditions of insulin resistance, NO is decreased, and this may cause hypertension [48].

This study has several strengths and should be noted. To the best of our knowledge, this is the first study to evaluate the mediatory role of inflammatory markers on the association of dietary insulin index and insulin load with metabolic syndrome in obese and overweight women. Furthermore, we tried to control for potential confounders in the analyses to yield a reliable result. However, this present study had some limitations that should be considered. Since this is a cross-sectional study, causal inferences cannot be made. In addition, the subjects of our research were overweight or obese women, and therefore our results should not be generalized to other populations. Furthermore, although we used a validated FFQ for dietary intake assessment, there might be some recall biases and misclassifications. Moreover, in the FFQ, we had some food items that were not in the insulin index database, and we had to use the values of similar foods, which may reduce the accuracy [23]. Finally, although we sought to control major confounders, the possibility of residual confounding bias remains because unknown or unmeasured confounders might have affected our findings.

## 5. Conclusion

In conclusion, significant associations were found between DII, DIL, and some components of MetS, including WC, TG, and SBP/DBP. A marginally significant positive association was found between DII and the risk of MetS. However, no association was seen between DIL and MetS. According to our study, two inflammatory markers (hs-CRP and MCP-1) had mediatory roles in MetS components. Moreover, in the association between DIL and MetS, both inflammatory markers had mediatory roles, but in the association between DII and MetS, only MCP-1 was seen to have a mediatory role.

## Figures and Tables

**Table 1:** General participant characteristics among median dietary insulin loads (DILs) and dietary insulin index (DII) in an obese and overweight woman (*n* = 219).

Variables	DII median		*P* value	*P* value^*∗*^	DIL median		*P* value	*P* value^*∗*^
	Low <38.82 *n* = 109	High ≥38.82 *n* = 110			Low <97155.69 *n* = 109	High ≥97155.69 *n* = 110		
	Mean (SD)	Mean (SD)			Mean (SD)	Mean (SD)		
Age (years)	35.37 (8.54)	36.49 (8.32)	0.32	0.22	36.30 (8.60)	35.56 (8.29)	0.52	0.21
Physical activity (MET-minutes/week)	1242.30 (1858.69)	1275.52 (2549.64)	0.91	0.45	1269.36 (2203.09)	1246.71 (2235.26)	0.94	0.32
DBP (mm Hg)	77.14 (10.12)	79.00 (9.72)	0.17	0.24	77.55 (9.30)	78.57 (10.57)	0.45	0.74
SBP (mm Hg)	110.86 (12.98)	112.21 (13.37)	0.46	0.94	110.50 (12.93)	112.55 (13.37)	0.26	0.80
Anthropometric variables								
Weight (kg)	79.75 (10.43)	78.62 (10.31)	0.42	0.35	78.34 (10.07)	80.06 (10.63)	0.22	0.50
Height (cm)	161.28 (5.39)	161.33 (5.89)	0.95	0.70	160.69 (5.56)	161.93 (5.65)	0.10	0.60
WC (cm)	93.84 (12.44)	96.05 (18.76)	0.37	0.61	92.96 (15.95)	97.00 (15.28)	0.10	0.38
BMI (kg/m^2^)	30.61 (3.70)	30.26 (3.45)	0.46	0.58	30.30 (3.44)	30.57 (3.71)	0.57	0.32
Biochemical parameters								
FBG (mg/dL)	86.74 (9.62)	86.72 (9.83)	0.98	0.93	86.64 (9.45)	86.82 (10.00)	0.89	0.93
TG (mg/dL)	119.14 (59.32)	119.48 (59.75)	0.96	0.20	121.05 (61.29)	117.53 (57.63)	0.66	0.24
HDL-c (mg/dL)	47.15 (9.55)	45.40 (11.43)	0.22	0.79	46.64 (11.24)	45.90 (9.84)	0.60	0.74
TC (mg/dl)	183.80 (35.55)	184.33 (36.07)	0.91	0.08	185.75 (39.05)	182.36 (32.12)	0.48	0.33
LDL-c (mg/dl)	95.12 (24.61)	92.50 (23.06)	0.42	0.66	93.37 (24.97)	94.24 (22.72)	0.79	0.21
hs-CRP (mg/l)	4.14 (4.58)	4.41 (4.55)	0.67	0.47	3.96 (4.39)	4.58 (4.75)	0.33	0.33
MCP-1 (mg/l)	57.85 (105.07)	43.83 (76.61)	0.29	0.31	49.29 (100.35)	52.87 (82.90)	0.79	0.42
HOMA-IR	3.37 (1.28)	3.37 (1.33)	0.99	0.45	3.48 (1.29)	3.27 (1.31)	0.23	0.60
Insulin (*μ*U/ml)	1.21 (0.23)	1.22 (0.23)	0.77	0.97	1.19 (0.22)	1.23 (0.24)	0.27	0.38
Body composition								
BFM (kg)	33.32 (7.24)	32.30 (6.86)	0.28	0.31	32.55 (6.68)	33.07 (7.44)	0.59	0.36
FFM (kg)	46.28 (5.20)	46.53 (5.54)	0.72	0.82	45.66 (5.43)	47.159 (5.20)	**0.03**	0.93

Categorical variables	*N* (%)	*N* (%)			*N* (%)	*N* (%)		

Economic level			**0.04**	0.33			0.10	0.79
Low level	33 (32.4)	21 (20.4)			32 (31.4)	22 (21.4)		
Moderate level	38 (37.3)	55 (53.4)			39 (38.2)	54 (52.4)		
High level	31 (30.4)	27 (26.2)			31 (30.4)	27 (26.2)		
Education level			**0.07**	0.24			0.16	**0.02**
Illiterate	0 (0.0)	3 (2.8)			0 (0.0)	3 (2.8)		
Under diploma	12 (11.0)	12 (11.2)			12 (10.9)	12 (11.3)		
Diploma	49 (45.0)	34 (31.8)			48 (43.6)	35 (33.0)		
Bachelor and higher	48 (44.0)	58 (54.2)			50 (45.5)	56 (52.8)		
Marital status			**0.05**	0.31			0.28	0.58
Single	24 (22.0)	36 (33.6)			27 (24.5)	33 (31.1)		
Married	85 (78.0)	71 (66.4)			83 (75.5)	73 (68.9)		

BFM: body fat mass, BMI: body mass index, DBP: diastolic blood pressure, DIL: dietary insulin load, DII: dietary insulin index, F BG: fasting blood glucose, FFM: fat-free mass, HDL_C: high-density lipoprotein cholesterol, hs-CRP: high-sensitivity c-reactive protein, HOMA-IR: homeostasis model-insulin resistance index, LDL_C: low-density lipoprotein cholesterol, MCP-1: monocyte chemoattractant protein-1, MET: metabolic equivalent, SBP: systolic blood pressure, TC: total cholesterol, TG: triglyceride, and WC: waist circumference. Values are represented as means (SD) or numbers (%). *P* values obtained from ANOVA or chi-square test. ^*∗*^*P* value reported after adjusting potential confounding factors (age, BMI, energy intake, and physical activity) using ANCOVA. BMI considers a collinear variable for anthropometrics and body composition variables. *P* values <0.05 were considered significant, *P* values 0.05–0.07 were considered as marginally significant. *P* values marked in bold show significant or marginally significant differences.

**Table 2:** Dietary intakes of the study population between the median of DII and DIL (*n* = 219).

Variables	DII median		*P* value	*P* value^∗^	DIL median		*P* value	*P* value^*∗*^
	Low <38.82 *n* = 109	High >38.82 *n* = 110			Low <97155.69 *n* = 109	High >97155.69 *n* = 110		
	Mean (SD)	Mean (SD)			Mean (SD)	Mean (SD)		
*Food groups*								
Whole grains (g/d)	8.30 (10.80)	7.98 (11.14)	0.82	0.60	7.59 (10.14)	8.68 (11.72)	0.46	0.44
Fruits (g/d)	456.94 (286.88)	619.14 (405.27)	**<0.001**	**0.01**	385.50 (247.91)	690.57 (389.09)	**<0.001**	0.88
Vegetables (g/d)	454.31 (260.71)	439.43 (273.50)	0.68	0.20	401.19 (248.33)	492.55 (277.49)	**0.01**	**0.04**
Legumes (g/d)	61.15 (46.53)	47.73 (39.35)	**0.02**	**<0.001**	49.20 (38.16)	59.69 (47.88)	**0.07**	0.58
Nuts (g/d)	17.02 (19.07)	14.96 (16.26)	0.39	0.05	12.29 (16.16)	19.69 (18.47)	**<0.001**	**0.02**
Dairy (g/d)	371.76 (244.18)	423.43 (270.36)	0.14	0.56	317.02 (204.36)	477.67 (281.37)	**<0.001**	0.95
Eggs (g/d)	21.13 (13.42)	21.71 (14.43)	0.75	0.87	18.85 (11.65)	23.98 (15.47)	**<0.001**	0.36
Meat (g/d)	70.51 (56.73)	60.30 (37.20)	0.11	**<0.001**	52.88 (32.11)	77.94 (57.50)	**<0.001**	0.35

*Energy and macronutrients*								
Energy (kcal)	2496.26 (771.18)	2742.51 (729.87)	**0.01**	—	2029.80 (392.70)	3208.9 (549.69)	**<0.001**	—
Carbohydrates (g/d)	335.66 (114.91)	411.01 (119.03)	**<0.001**	**<0.001**	279.72 (63.05)	466.9 (92.59)	**<0.001**	**0.01**
Fat (g/d)	97.45 (35.55)	91.08 (29.28)	0.15	**<0.001**	76.04 (24.59)	112.49 (29.44)	**<0.001**	**<0.001**
Protein (g/d)	86.54 (30.29)	91.48 (25.18)	0.19	0.25	71.22 (17.97)	106.80 (24.54)	**<0.001**	**<0.001**
Total fiber (g/d)	44.45 (20.63)	45.52 (16.98)	0.67	0.17	36.03 (15.74)	53.93 (17.47)	**<0.001**	0.16
PUFA (g/d)	21.74 (9.93)	18.04 (6.75)	**<0.001**	**<0.001**	17.430 (8.74)	22.355 (7.91)	**<0.001**	**<0.001**
SFA (g/d)	27.61 (11.86)	28.66 (10.65)	0.49	0.08	21.834 (7.21)	34.442 (11.07)	**<0.001**	0.91

*Micronutrients*								
Iron (mg/d)	17.42 (5.97)	19.98 (5.83)	**<0.001**	**<0.001**	14.34 (3.57)	23.06 (4.68)	**<0.001**	0.13
Zinc (mg/d)	12.29 (4.28)	13.94 (4.15)	**<0.001**	**0.06**	10.14 (2.78)	16.09 (3.36)	**<0.001**	0.08
Calcium (mg/d)	1109.28 (439.15)	1235.43 (406.79)	**0.02**	0.27	937.00 (330.86)	1407.71 (380.91)	**<0.001**	0.54
Magnesium (mg/d)	430.30 (145.49)	500.58 (144.78)	**<0.001**	**<0.001**	366.66 (115.44)	564.22 (107.72)	**<0.001**	0.26
Potassium (mEq/d)	4160.62 (1544.47)	4641.50 (1594.98)	**0.02**	0.36	3489.26 (1206.51)	5312.86 (1382.83)	**<0.001**	0.58
Sodium (mg/d)	4194.90 (1455.36)	4291.66 (1432.26)	0.62	0.20	3504.49 (983.78)	4982.08 (1450.73)	**<0.001**	0.88
Phosphorus (mg/d)	1541.34 (532.46)	1754.66 (490.52)	**<0.001**	**0.01**	1296.81 (350.49)	1999.19 (418.68)	**<0.001**	**0.07**
Vitamin C (mg/d)	172.33 (102.26)	221.02 (151.95)	**<0.001**	**0.06**	142.75 (83.86)	250.59 (147.79)	**<0.001**	0.72
Vitamin E (mg/d)	18.59 (10.57)	15.41 (6.77)	**<0.001**	**<0.001**	15.26 (8.71)	18.74 (8.98)	**<0.001**	**0.03**
Thiamin (mg/d)	1.86 (0.629)	2.26 (0.59)	**<0.001**	**<0.001**	1.58 (0.37)	2.54 (0.46)	**<0.001**	**<0.001**
Riboflavin (mg/d)	2.09 (0.81)	2.35 (0.86)	**0.02**	0.24	1.73 (0.53)	2.71 (0.82)	**<0.001**	0.37
Niacin (mg/d)	24.12 (9.32)	26.16 (8.25)	0.08	0.91	19.84 (5.18)	30.44 (8.57)	**<0.001**	0.86
Vitamin B6 (mg/d)	2.05 (0.74)	2.30 (0.66)	**<0.001**	0.12	1.72 (0.468)	2.62 (0.63)	**<0.001**	0.72
Folate (mcg/d)	572.63 (176.35)	640.46 (168.76)	**<0.001**	0.08	491.786 (123.818)	721.31 (141.37)	**<0.001**	0.55
Vitamin B12 (mcg/d)	4.63 (3.15)	4.40 (1.73)	0.50	**0.07**	3.61 (1.57)	5.42 (2.96)	**<0.001**	0.97

DIL: dietary insulin load, DII: dietary insulin index, PUFA: polyunsaturated fatty acid, and SFA: saturated fatty acid. Values are represented as means (SD). *P* values obtained from ANOVA. ^*∗*^*P* value obtained from ANCOVA reported after adjusting total energy intake. *P* values <0.05 were considered as significant. *P* values 0.05–0.07 are considered as marginally significant. *P* values marked in bold show significant or marginally significant differences.

**Table 3:** Association between MetS and its components with DIL and DII in obese and overweight women (*n* = 219).

Variables		DII median			DIL median		
		OR	95% CI	*P* value	OR	95% CI	*P* value
WC (cm)	Crude	1.21	0.58, 2.53	0.60	0.94	0.45, 1.96	0.88
	Model 1	1.46	0.63, 3.41	0.37	1.07	0.31, 3.65	0.90
	Model 2	1.67	1.09, 4.03	**0.03**	1.27	0.34, 4.72	0.71

TG (mg/dL)	Crude	0.83	0.44, 1.56	0.56	0.83	0.44, 1.56	0.56
	Model 1	1.26	0.61, 2.61	0.51	1.28	0.42, 3.86	0.65
	Model 2	1.10	0.92, 2.33	**0.07**	1.20	0.38, 3.84	0.74

HDL-C (mg/dL)	Crude	1.10	0.62, 1.93	0.73	1.07	0.61, 1.88	0.81
	Model 1	0.98	0.52, 1.84	0.95	0.77	0.29, 2.04	0.59
	Model 2	1.15	0.59, 2.23	0.67	0.81	0.28, 2.33	0.70

FBG (mg/dL)	Crude	1.14	0.39, 3.27	0.80	1.16	0.40, 3.33	0.77
	Model 1	0.56	0.16, 1.92	0.36	0.58	0.09, 3.71	0.56
	Model 2	0.65	0.18, 2.39	0.52	0.66	0.08 5.01	0.69

SBP (mm Hg)	Crude	1.53	0.52, 4.53	0.43	1.41	0.54, 3.68	0.47
	Model 1	1.14	0.38, 3.39	0.81	0.50	0.09, 2.78	0.43
	Model 2	1.25	0.38, 4.07	0.70	0.52	0.08, 3.46	0.50

DBP (mm Hg)	Crude	1.88	0.95, 3.71	**0.06**	1.79	0.90, 3.53	0.09
	Model 1	1.59	0.75, 3.37	0.22	2.09	0.67, 6.50	0.19
	Model 2	1.54	0.70, 3.42	0.28	2.05	0.61, 6.87	0.24

SBP/DBP (mmHg)	Crude	2.12	1.10, 4.11	**0.02**	1.62	0.84, 3.10	0.14
	Model 1	1.71	0.82, 3.55	0.14	1.58	0.53, 4.74	0.40
	Model 2	1.84	0.85, 3.99	**0.07**	1.74	1.54, 5.61	**0.04**

MetS	Crude	1.56	0.78, 3.12	0.20	1.13	0.57, 1.21	0.71
	Model 1	1.63	0.76, 3.51	0.20	1.06	0.35, 2.65	0.90
	Model 2	2.11	0.93, 4.82	**0.06**	1.59	0.21, 2.21	0.45

CI: confidence interval, DBP: diastolic blood pressure, DIL: dietary insulin load, DII: dietary insulin index, FBG: fasting blood glucose, HDL_C: high-density lipoprotein cholesterol, MetS: metabolic syndrome, OR: odds ratio, SBP: systolic blood pressure, TG: triglyceride, and WC: waist circumference. Binary logistic regression was used. The lower median of DIL (<97155.69) and DII (>38.82) is considered a reference group. Model 1: adjusted for age, BMI, energy intake, and physical activity (BMI consider a collinear variable). Model 2: model 1+ education, marital, and economic status. *P* values <0.05 were considered significant. *P* values 0.05–0.07 are considered marginally significant. *P* values marked in bold show significant or marginally significant association.

**Table 4:** MetS and its components among DIL and DII in obese and overweight women (*n* = 219).

Variables		DII median			DIL median		
		OR	95% CI	*P* value	OR	95% CI	*P* value
WC (cm)	hs_CRP	1.65	0.65, 4.14	0.28	1.19	0.30, 4.71	0.80
	MCP-1	1.44	0.57, 3.65	0.43	1.47	0.23, 9.24	0.67

TG (mg/dL)	hs_CRP	1.23	0.55, 2.71	0.60	1.27	0.37, 4.39	0.69
	MCP-1	1.34	0.57, 3.10	0.49	1.57	0.44, 5.61	0.48

HDL-c (mg/dL)	hs_CRP	1.12	0.57, 2.22	0.73	0.67	0.23, 1.99	0.47
	MCP-1	0.99	0.49, 2.00	0.98	0.93	0.30, 2.82	0.90

FBG (mg/dL**)**	hs_CRP	0.50	0.10, 2.33	0.37	0.43	0.03, 5.53	0.52
	MCP-1	0.73	0.18, 2.95	0.66	0.37	0.04, 3.28	0.37

SBP (mmHg)	hs_CRP	1.20	0.35, 4.04	0.76	0.44	0.06, 3.14	0.41
	MCP-1	1.59	0.34, 7.36	0.54	0.45	0.04, 4.84	0.51

DBP (mmHg)	hs_CRP	1.56	0.65, 3.69	0.31	1.83	0.49, 6.81	0.36
	MCP-1	1.95	0.76, 4.98	0.16	2.96	0.73, 11.99	0.12

SBP/DBP (mmHg)	hs_CRP	1.88	0.82, 4.33	0.13	1.49	0.42, 5.28	0.53
	MCP-1	2.43	0.98, 5.99	0.05	2.33	0.622, 8.75	0.20

MetS	hs_CRP	0.38	0.15, 0.96	0.03	0.63	0.16, 2.40	0.50
	MCP-1	2.08	0.83, 5.20	0.11	1.53	0.39, 5.87	0.53

CI: confidence interval, DBP: diastolic blood pressure, DIL: dietary insulin load, DII: dietary insulin index, FBG: fasting blood glucose, HDL_C: high-density lipoprotein cholesterol, hs-CRP: high-sensitivity c-reactive protein, MetS: metabolic syndrome, MCP-1: monocyte chemoattractant protein-1, OR: odds ratio, SBP: systolic blood pressure, TG: triglyceride, and WC: waist circumference. Binary logistic regression was used. The lower median of DIL (<97155.69) and DII (<38.82) consider a reference group. hs_CRP: adjusted for age, energy intake, physical activity, education status, marital status, economic status, and hs_CRP. MCP-1: adjusted for age, energy intake, physical activity, education status, marital status, economic status, and MCP-1.

## Data Availability

The datasets used and/or analyzed during the current study are available from the corresponding author on reasonable request.
